# Impact of praziquantel treatment and early reinfection patterns in a mixed *Schistosoma mansoni* and *Schistosoma haematobium* infection focus in the Democratic Republic of Congo

**DOI:** 10.1371/journal.pntd.0014764

**Published:** 2026-10-09

**Authors:** Joule Ntwan Madinga, Clementine Roucher, Sylvie Linsuke, Sylvain Baloji, Marie Hermy, Pascal Lutumba, Katja Polman

**Affiliations:** 1 Institut National de Recherche Biomédicale, Kinshasa, Democratic Republic of the Congo; 2 Université de Kikwit, Kikwit, Democratic Republic of the Congo; 3 Institute of Tropical Medicine of Antwerp, Antwerp, Belgium; 4 Programme National de Lutte contre la Trypanosomiase Humaine Africaine, Kinshasa, Democratic Republic of the Congo; 5 Université de Kinshasa, Kinshasa, Democratic Republic of the Congo; Seoul National University College of Medicine, KOREA, REPUBLIC OF

## Abstract

**Objective:**

To evaluate the impact of single versus mixed Schistosoma infections on the efficacy of PZQ treatment as well as on the dynamics of reinfection after treatment.

**Methods:**

We followed 388 randomly selected residents of a village co‑endemic for *Schistosoma haematobium* and *S. mansoni*, screening them for *Schistosoma* infection every three weeks across a nine‑week period. Kato-Katz stool examination was used to detect eggs of *S. mansoni* and urine filtration to detect eggs of *S. haematobium*. At every visit, those who were positive for schistosomiasis were treated with 40mg/kg praziquantel. Treatment outcomes (cure rate (CR), egg reduction rate (ERR), cumulative cure rate (CCR) and cumulative egg reduction rate (CERR)) and reinfection rates were compared between age groups, sex and pre-treatment co-infection status using McNemar and Wilcoxon tests.

**Results:**

Baseline infection prevalence was similar for *S. mansoni* (60.8%) and *S. haematobium* (60.3%), with 36.6% of mixed *S. haematobium*-*S. mansoni* infection. Infection intensity was higher in mixed compared to single infection (200.7 versus 70 epg for *S. mansoni*, p < 0.001; 29 versus 9.6 ep10mL for *S. haematobium*, p < 0.001). After one praziquantel treatment, CR of 62.7% and 94.6%, with ERR of 97.5% and 96.5%, were found for *S. haematobium* and *S. mansoni*, respectively. After 3 rounds of praziquantel treatment, CCR of 75% and 93.7%, with CERR of 95.6% and 97.5%, were recorded for *S. haematobium* and *S. mansoni*, respectively. In addition, treatment efficacy was significantly higher in single than in mixed infections (CR = 76.7% versus 58%, p = 0.01; ERR = 90.2% versus 96.5%, p < 0.001; CCR = 76.7% versus 58%, p = 0.01; CERR = 90.2% versus 96.5%, p < 0.001), and in older than in younger (<16 years) age groups. No significant associations were found between (co-)infection status and reinfection rate.

**Conclusion:**

Our results show that pre-treatment co-infection status can affect schistosomiasis treatment outcomes for *S. haematobium* but does not affect the post-treatment reinfection. These results could help in tailoring control activities in co-endemic areas.

## Author summary

Schistosomiasis is a neglected tropical disease caused by parasitic worms that infect more than 250 million people worldwide, particularly in sub-Saharan Africa. In many endemic communities, people are simultaneously infected with both *Schistosoma haematobium* and *Schistosoma mansoni*, yet little is known about whether these mixed infections influence the effectiveness of praziquantel, the only widely used drug for schistosomiasis control. We followed residents of a co-endemic village before and after repeated praziquantel treatment to compare treatment response and reinfection between individuals with single-species and mixed-species infections. We found that mixed infections were associated with substantially higher parasite burdens than single infections. Individuals with mixed infections, particularly those infected with *S. haematobium*, responded less well to treatment than those with single infections, whereas treatment efficacy improved with increasing age. In contrast, mixed infections did not increase the risk of becoming reinfected after treatment. These findings suggest that the presence of mixed schistosome infections should be considered when evaluating treatment outcomes and planning control programmes in co-endemic areas. Tailoring treatment and monitoring strategies to account for mixed infections could improve the effectiveness of schistosomiasis control efforts.

## Introduction

Schistosomiasis is a parasitic disease caused by trematode worms of the genus *Schistosoma* [1]. Approximately 240 million people are affected worldwide with 90% reported in Sub Saharan Africa [2]. The major species prevailing in SSA are *Schistosoma mansoni*, agent of intestinal schistosomiasis and *S. haematobium*, agent of urogenital schistosomiasis. This disease is prevalent in tropical and subtropical areas where hygiene standards are poor and water bodies infested with *Schistosoma* intermediate fresh water snail hosts. The global distribution map of *Schistosoma* shows a large overlap in many areas of *S. mansoni* and *S. haematobium* endemic areas in Africa, with many people being at risk of co-infection with both species [3]. Yet, little is known about the epidemiology and control of Schistosoma mixed infections.

Schistosomiasis control focuses on reducing morbidity through preventive chemotherapy, with periodic large-scale treatment of at-risk groups, mainly school aged children, using praziquantel (40 mg/Kg) [4]. The performance of preventive chemotherapy is commonly assessed in the literature terms of cure rates (CR) and egg reduction rates (ERR), though WHO recommends using ERR as the indicator of choice for drug efficacy assessment, and considers ERR of ≥ 90% satisfactory [5]. Praziquantel is effective against all species of Schistosoma, although some studies have reported a higher efficacy against one species than another [6]. Factors affecting praziquantel efficacy include parasite strain/genotype [7], parasite development stage susceptibility [8], drug quality or bioavailability [9], and host-related factors such as age and higher initial infection intensity [10]. Without control measures tackling schistosomiasis transmission, successful treatment is followed by rapid reinfection. Host determinants of reinfection with *Schistosoma* include pretreatment positive status, high pretreatment infection intensity, younger age, malnutrition or stunting, poorer household asset or living in high endemic area [11,12]. Despite the established association between mixed infection and high infection intensity, and between the latter and reinfection, very few studies have assessed the impact of anthelminthic treatment on mixed infection and the effect of pre-treatment mixed infection status on subsequent reinfection dynamics [13–15].

The Democratic Republic of the Congo (DRC) is endemic for both urogenital and intestinal schistosomiasis [16]. It is one of the World Health Organization (WHO) priority countries in the efforts against schistosomiasis. Despite a scale-up of mass drug administration (MDA) since 2015, there were still 10–15 million children in need of praziquantel treatment in 2023 [17].The current study aimed to assess efficacy of praziquantel treatment on single versus mixed Schistosoma infections as well as the impact of pre-treatment mixed infection status on the dynamics of reinfection after treatment. Such knowledge would be of great interest for surveillance, treatment, and schistosomiasis control in co-endemic settings.

## Methods

### Ethics statement

The study was conducted in accordance with the principles of the Declaration of Helsinki. Ethical approval was obtained from the Institutional Review Board of the Institute of Tropical Medicine, Antwerp (No. 852/12), and the Ethical Committee of the University of Antwerp (No. 12/50/423). In the Democratic Republic of the Congo, the study protocol was approved by the Ethics Committee of the University of Kinshasa (ESP/CE/050/2014), and authorization was granted by the Ministry of Health. Prior to data collection, permission was obtained from local administrative and traditional authorities. Community leaders organized sensitization meetings during which the study objectives, procedures, participants’ rights, and the voluntary nature of participation were explained in detail. Written informed consent was obtained from all adult participants. For participants under 18 years of age, written consent was obtained from a parent or legal guardian. Participants who tested positive for schistosomiasis were treated with praziquantel (40 mg/kg) according to national treatment guidelines.

### Study area and population

The field study was conducted from March to June 2016 in Kifwa II (5°33’S and 4°21’E), a village situated in the rural health district of Kwilu-Ngongo, Kongo central (former Bas-Congo) province, in western DRC. Situated at 125 Km from Kinshasa, the capital city, on the national paved road, the village lies along “Ngongo” river and water supply depends on the proximity to different water points (river, artisanal water wells, etc.). At the time of the study the village counted around 1,300 inhabitants, mostly farmers. Kongo central province, as many other rural areas of the DRC is characterized by poor hygiene standards [18] and many areas of this province are known to be endemic for schistosomiasis [16,19]. Prior to the current study, no other schistosomiasis control activities had taken place in Kifwa II.

### Study design and data collection

The current study is part of a larger community-wide study on the epidemiology of helminth co-infections and associated diseases [20]. This sub-study was designed as a cohort study following up a random subsample of individuals that participated to the baseline community-wide study. The cohort was followed up during nine weeks at three-week intervals with four parasitological surveys at baseline, week 3, 6 and week 9. At each visit, two stool and two urine samples were requested from each participant. Individual demographic (age and sex) data were collected using a questionnaire. The flow diagram of the sub-study is presented in the Fig 1.

**Fig 1:**
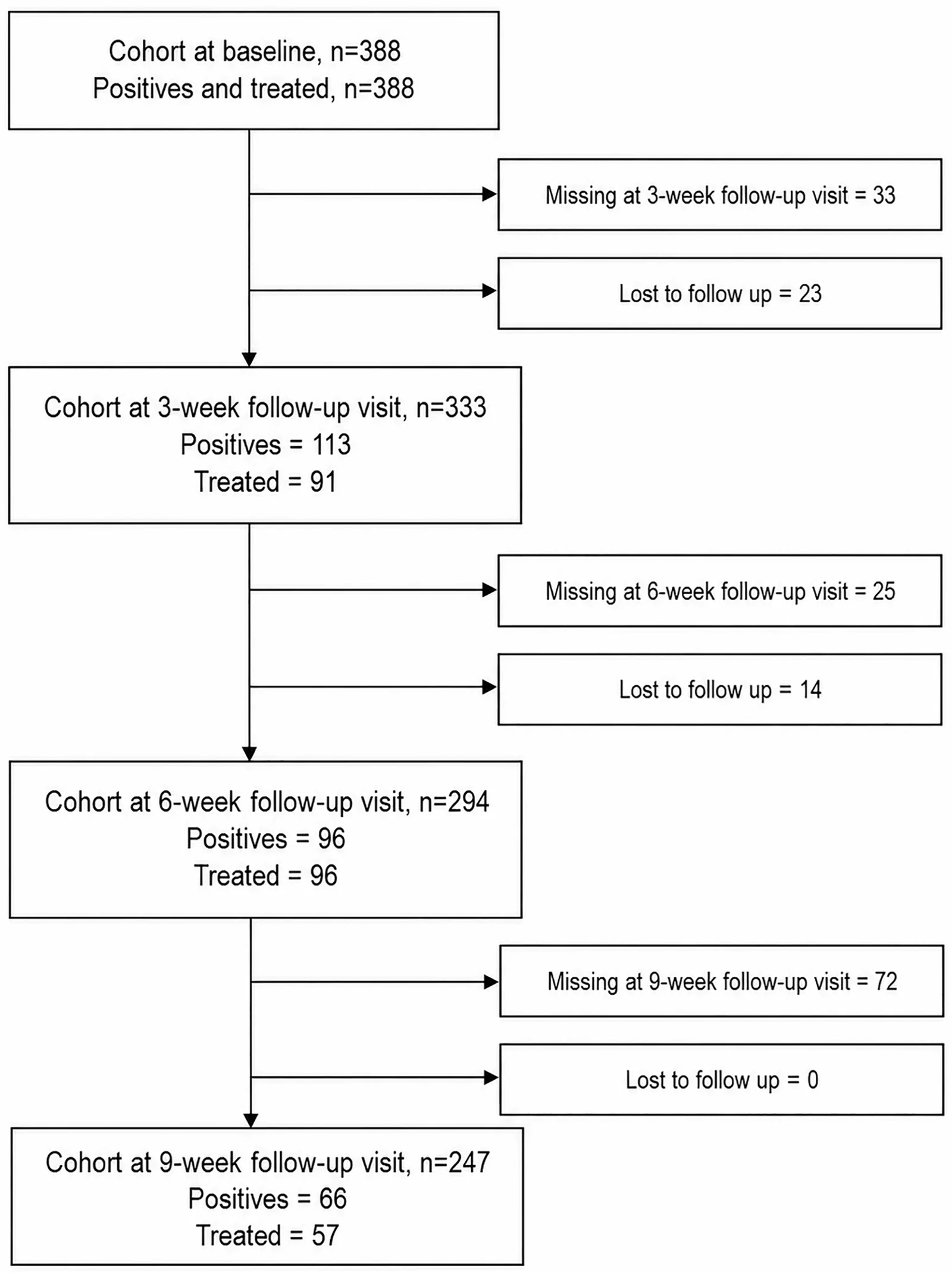
Flow diagram of participant follow-up from baseline to the 9-week visit.

A total of 388 participants were enrolled and treated at baseline. At the 3-week follow-up visit, 33 were missing and 23 were lost to follow-up, leaving 333 participants, of whom 113 tested positive and 91 were treated. At the 6-week visit, 25 were missing and 14 lost to follow-up, with 294 participants remaining (96 positives, all treated). At the 9-week visit, 72 participants were missing, and 247 were followed up (66 positives, 57 treated).

### Infection and treatment

All collected samples were kept in a cool box with ice packs (+/-4°C) and brought to the laboratory of the Medical Evangelical Institute (IME) of Kimpese for parasitological analysis. For each feces sample, two Kato-Katz slides of 25 mg of fecal material each were prepared to detect *S. mansoni* eggs by microscopy [21]. Urine samples were tested for the presence of *S. haematobium* eggs using microscopy following a urine filtration technique of 10 ml of urine [22]. At each visit, participants who tested positive for schistosomiasis were treated with 40 mg/Kg. Tablets were swallowed under direct observation of the nurse or the community health workers. For children, the pills were crushed in a spoon and mixed with water.

### Statistical analysis

Data were doubly entered using Microsoft Access 2011 and analyzed using R studio. Baseline infection characteristics were described as percentage of positives and infection intensity by species and stratified by demographic characteristics and pre-treatment single/mixed infection status. Infection intensity was expressed as arithmetic mean of egg count per 10 mL (ep 10ml) of urine for *S. haematobium* and egg count per gram (epg) of stool for *S. mansoni* infection. Patterns of infection over time were described as percentage of infection by Schistosoma species stratified by demographic characteristics and pre-treatment mixed infection status.

#### Efficacy of anthelminthic treatment.

The efficacy of anthelminthic treatment was assessed in two ways: the effect of a single dose of anthelminthic treatment assessed at week 3 and the cumulative effect of multiple treatments assessed at week 9. The following index were calculated:

i CR: percentage of Schistosoma-positives found to be negative after treatment between two measurement times, calculated as CR= [1 - (# positives after treatment/#positives before treatment)]*100%;
ii ERR: percentage reduction in arithmetic mean after treatment, between two measurement time, calculated as ERR= [1 - (arithmetic mean egg count after treatment/ arithmetic mean egg count at baseline)]*100%;
iii CCR: the total percentage of Schistosoma positives at baseline cured after multiple rounds of treatment, calculated in participants present from baseline until the 9-week follow-up visit;
iv CERR: the total percentage reduction in arithmetic mean after multiple rounds of treatment, calculated in participants present from baseline until the 9-week follow-up visit.

#### Reinfection patterns.

Reinfection was defined as a participant who reverted to a positive test for the same Schistosoma species after having become negative at a previous follow‑up visit. This includes:

(i) Participants that were positive for any Schistosoma species at the baseline survey, became negative at the 3-week follow-up visit and reverted to positive with the same Schistosoma species either at the 6 or 9-week follow-up visit;
(ii) Participants that were positive for any Schistosoma species at the 3-week follow-up visit, became negative at the 6-week follow-up visit and reverted to positive with the same Schistosoma species at 9-week follow-up visit.

Pearson’s CHI squared or Fisher’s exact test were used to test differences of proportions between categories of participants’ characteristics (age, sex and pre-treatment single/mixed infection status) at baseline. McNemar test was used for proportion comparison between time points (paired data). Wilcoxon test was used to assess differences of ERR and CERR between categories of participants characteristics. Age was categorized in <16 years old (the target age-group for MDA programs in the DRC) and ≥ 16 years old.

## Results

### Study population

A total of 388 participants were enrolled at baseline, including 202 (52%) female and 186 (48%) male. Age ranged from 1 to 80 years with a median of 14 years (IQR = 8–36 years). Of these, 247 (63.6%) participated in all follow-up visits.

### Baseline Schistosoma infection characteristics

Table 1 presents the prevalence and infection intensity by *Schistosoma* species stratified by demographical characteristics and pre-treatment infection status. Baseline prevalence was similar for both species (60.3% for *S. haematobium* and 60.8% for *S. mansoni*). Furthermore, the prevalence of *S. haematobium* was significantly higher in females compared to males (64.4% versus 55.9%, p = 0.04) and in participants under 16 years of age compared to those aged 16 years and above (71.6% versus 48.7%, p < 0.001). A total of 142 (36.6%) participants harbored mixed infection. Mixed infection prevalence was higher in participants under 16 years of age compared to those aged 16 years and above (46.2% versus 26.7%, p < 0.001).

**Table 1:** Baseline helminth infection characteristics.

Factors	N	*Schistosoma haematobium*				*Schistosoma mansoni*				Mixed infection	
		Positive, n(%)	p	Egg count (ep10mL)	P	Positive, n(%)	p	Egg count (epg)	p	Positive, n(%)	p
Overall	388	234 (60.3)		16.8		236 (60.8)		118		142 (36.6)	
**Age category**											
<16 years	197	141 (71.6)	<0.001	21.5	<0.01	125 (63.4)	0.3	127.2	0.14	91 (46.2)	<0.001
≥16 years	191	93 (48.7)		11.9		111 (58.1)		108.6		51 (26.7)	
**Sex**											
M	186	104 (55.9)	0.04	14.7	0.13	119 (64)	0.13	128.5	0.13	69 (37.1)	0.42
F	202	130 (64.4)		18.7		117 (57.9)		108.4		73 (36.1)	
**Mixed infection**											
No	246	92 (37.4)	0	9.6	<0.001	94 (38.2)	0	70	<0.001	NA	NA
Yes	142	142 (100)		29		142 (100)		200.7		NA	

Overall infection intensity was 16.8 ep10mL and 118 epg for *S. haematobium* and *S. mansoni* respectively. Infection intensity was higher in participants with mixed infection for both S. haematobium and S. mansoni (29 versus 9.6 ep10mL; 200.7 versus 70 epg, p < 0.001). In addition, *S. haematobium* infection intensity was higher in the younger (<16 years) than in the older (≥ 16 years) age group (21.5 versus 11.9 ep10mL, p = 0.01) (Table 1 here).

### Schistosoma infection characteristics over time

Overall, there was a downward trend in the frequency of single and mixed Schistosoma infections over time, with a slight increase at the 6-week follow-up visit (Fig 2). Infection intensity displayed also displayed a downward trend but the increase at the 6-week follow-up visit was only recorded among participants with mixed infections. At baseline, infection intensity was higher in mixed infections than in single infection. Conversely, at the 9-week follow-up visit, infection intensity was higher in participants with single *S. haematobium* infection compared to those with mixed infection.

**Fig 2:**
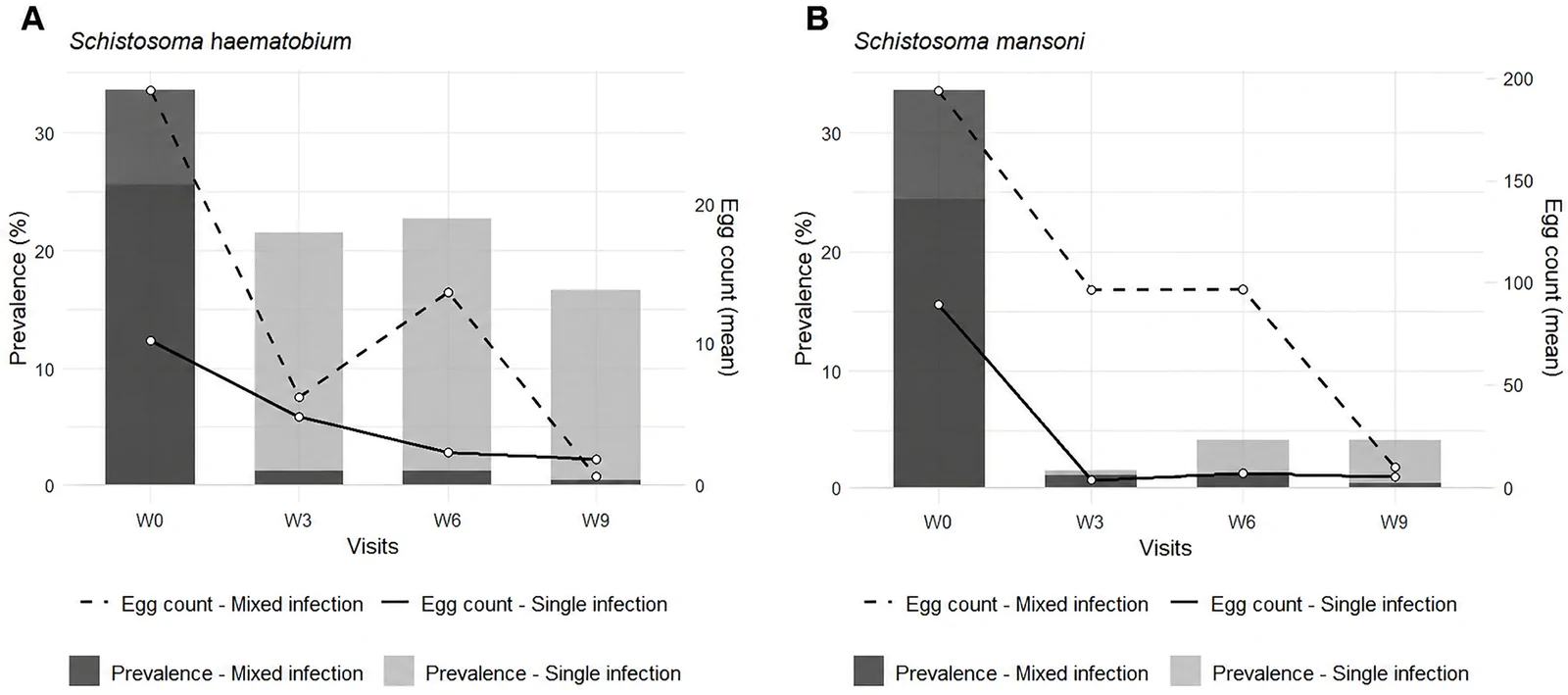
Prevalence of *Schistosoma haematobium* (A) and *Schistosoma mansoni* (B) positive participants and infection intensity by infection status over time in the cohort of inhabitants of Kifwa II village who participated in all follow-up rounds. Bars represent prevalence (%) for single (grey) and mixed (black) infections, while lines represent mean egg counts for each infection category at different visits (W0, W3, W6, W9), with full lines for single infections and dashed lines for mixed infections. Both the prevalence and intensity of single and mixed *Schistosoma* infections decreased over time, with a slight increase at the 6-week follow-up (W6). Infection intensity was higher in mixed infections at baseline but reversed by the 9-week follow-up (W9), with participants having single *S. haematobium* infections showing greater intensity than those with mixed infections.

### Treatment efficacy

Table 2 presents the results of treatment efficacy (CR and ERR) at week 3. CR were 62.7% for S. haematobium and 94.6% for *S. mansoni*. ERR were 97.5% for *S. haematobium* and 96.5% for *S. mansoni*. For *S. haematobium*, both CR and ERR were significantly higher in single infection compared to mixed infection (CR = 74.7% versus 54.9%, p = 0.003; ERR = 95.8% versus 79.3%, p = 0.01). In addition, CR and ERR were higher in the older (≥16 years) than in the younger (<16 years) age group (CR = 75% versus 55.2%, p = 0.003; ERR = 91.6% versus 82, p = 0.003). No significant differences in CR or ERR were found for *S. mansoni*. (Table 2 here).

**Table 2:** Cure rates and egg reduction rates in single and mixed infections with *Schistosoma spp.*

Factors	*Schistosoma haematobium*					*Schistosoma mansoni*				
	Positive (N)	Cured, n(%)	p	ERR (%)	p	Positive (N)	Cured, n(%)	P	ERR (%)	p
Overall	201	126 (62.7)		97.5		203	192 (94.6)		96.5	
**Age category**										
<16 years	125	69 (55.2)	0.003	82	0.003	110	103 (93.6)	0.37	96.7	0.98
≥16 years	76	57 (75)		91.6		93	89 (95.7)		96.3	
**Sex**										
M	89	53 (59.5)	0.25	77	0.7	100	94 (94)	0.48	95	0.77
F	112	73 (65.2)		92.6		103	98 (95.1)		98	
**Mixed Schistosoma infection**										
No	79	59 (74.7)	0.003	95.8	0.01	81	78 (96.3)	0.29	96.4	0.74
Yes	122	67 (54.9)		79.3		122	114 (93.4)		96.6	

Table 3 shows CCR and CERR after 3 rounds of treatment in those who were tested positive at the baseline and tested subsequently at all follow-up visits (n = 146). CCR was 75% for *S. haematobium* and 93.7% for *S. mansoni*; CERR was 95.6%for *S. haematobium* and 97.5%for *S. mansoni*. For *S. haematobium,* CCR was significantly higher among participants with single infection compared to those with mixed infection (82.5% versus 68.7%, p = 0.04). In addition, both CCR and CERR were significantly higher in the younger (<16 years) than in the older (≥16 years) age group (CCR = 84.9% versus 68.8%, p = 0.02; CERR = 96.5% versus 95.1%, p = 0.04). No significant differences in CCR or CERR were observed for *S. mansoni*.

**Table 3:** Cumulative cure rates and cumulative egg reduction rates in single and mixed infections with *Schistosoma* spp.

Factors	Schistosoma haematobium					Schistosoma mansoni				
	Positive (N)	Cured, n(%)	p	CERR (%)	p	Positive (N)	Cured, n(%)	P	CERR (%)	p
Overall	146	109 (75)		95.6		143	134 (93.7)		97.5	
**Age category**										
<16 years	93	61 (68.8)	0.02	95.1	0.04	77	71 (92.2)	0.3	99.2	0.5
≥16 years	53	45 (84.9)		96.5		65	62 (95.4)		95.4	
**Sex**										
M	64	46 (71.9)	0.3	96	0.54	72	67 (93.1)	0.52	99.5	0.83
F	82	63 (76.8)		95		70	66 (94.3)		95.3	
**Mixed Schistosoma infection**										
Single	63	52 (82.5)	0.04	96.9	0.05	60	55 (91.7)	0.29	95.5	0.39
Mixed	83	57 (68.7)		94.6		83	79 (95.2)		98.9	

### Patterns of reinfection

Overall, we found a reinfection rate of 26% for *S. haematobium* and 8.4% for *S. mansoni* (Table 4). The *S. haematobium* reinfection rate was significantly higher in the younger (<16 year) than the older (≥16 years) age group (36.4% versus 14%, p = 0.003).

**Table 4:** Proportion of reinfection in single and mixed infections with Schistosoma spp.

Factors	*Schistosoma haematobium*			*Schistosoma mansoni*		
	Cured (N)	Reinfected, n(%)	p	Cured (N)	Reinfected, n(%)	p
Overall	116	31 (26.7)		142	12 (8.4)	
**Age category**						
<16 years	66	24 (36.4)	0.03	76	7 (9.2)	0.49
≥16 years	50	7 (14)		66	5 (7.7)	
**Sex**						
M	48	13 (27.1)	0.5	72	9 (12.5)	0.07
F	68	18 (26.5)		70	3 (4.3)	
**Mixed Schistosoma infection**						
No	56	16 (28.6)	0.4	60	3 (5)	0.16
Yes	60	15 (25)		82	9 (11)	

## Discussion

Little is known about the impact of mixed *Schistosoma* infection on anthelminthic treatment performance and reinfection patterns after treatment. Such knowledge can bring more insight in the sustainability of preventive chemotherapy. The current study aimed to assess patterns of single and mixed *Schistosoma* infection before and after treatment with praziquantel.

Mixed *Schistosoma* infection was common in our study area, which is in line with other studies conducted in *S. mansoni - S. haematobium* co-endemic areas across SSA (see [23,24] for review). In addition, baseline *Schistosoma* infection intensity was significantly higher in people carrying mixed infection compared to those with single *Schistosoma* infections (29 versus 9.6 ep10ml, p < 0.001 and 200.7 versus 70 epg, p < 0.001). This higher infection intensity is suggestive of increased morbidity among people carrying both species and calls for public health interventions targeting this group.

Over time, both infection prevalence and infection intensity decreased, with a slight rise at the 6-week follow-up visit. This peak can be due to the occurrence of early reinfection in this high transmission area for schistosomiasis. It may also be due to egg production by worms that survived the first treatments [25]. Since praziquantel has only minor activity against the larval stage of schistosomes, these immature parasites can survive and grow up to be egg-producing adult worms.

While baseline prevalence for *S. mansoni* (60.3%) and *S. haematobium* (60.8%) was similar before treatment, *S. haematobium* appeared to be more prevalent than *S. mansoni* at 3 (22.7% vs 2.8%), 6 (23.9% vs 5.3%), and 9-week (17% vs 4.4%) follow-up visits. A similar alteration of the composition of *Schistosoma* species in a context of praziquantel treatment has been reported by Knowles et al.[14]. They explained this by competitive interactions occurring between *S. mansoni* and *S. haematobium* in the context of drug treatment, leading to emergence of *S. haematobium* in the period after treatment. In addition, while baseline infection intensity was higher in mixed *Schistosoma* compared to single infections (29 ep10mL versus 9.6 ep10mL, p < 0.001 and 200.7 epg versus 70 epg, p < 0.001), a shift was observed at the 9-week follow-up visit, with *S. haematobium* infection intensity turning higher in single than in mixed infections (0.5 ep10mL versus 1.8 ep10mL, p < 0.001). This reversion of infection intensity at 9-week follow-up visit could suggest that multiple treatments by reducing infection intensity could have removed the discrepancies between mixed and single infections.

We measured the effect of single treatment and the cumulative effect of multiple treatment. In both cases, ERR were satisfactory in regard of WHO criteria [5]. However, the CR against *S. haematobium* was consistently lower than for *S. mansoni* (62.7% versus 94.6% and 75% versus 93.7%, respectively). This discrepancy could possibly be explained by tolerance of the local *S. haematobium* strain [26]. However, since no MDA campaign with praziquantel had taken place in Kifwa II before our study, this hypothesis is less likely. Another explanation could be the difference in sensitivity between Kato Katz and urine filtration, and the extent to which this sensitivity declines after treatment [27,28]. While microscopy methods (Kato-Katz and urine filtration) become less sensitive after treatment, parasite eggs are easier to find in urine than in stool sample.

When comparing treatment outcomes by mixed infection status, CR and ERR on *S. haematobium* infection were significantly lower for people who had *Schistosoma* mixed infection at baseline than for those with single *S. haematobium* infection (74.7% versus 54.9%, p = 0.03 and 95.8% versus 79.3%, p = 0.01, respectively). Our data are in line with findings from the meta-analysis by Zwang et al [6] who also concluded that mixed infections are harder to clear. This can be explained by the higher pretreatment infection intensity found in participants with mixed *Schistosoma* infection. Indeed, infection intensity is inversely associated with CR and ERR [29,30]. However, it is not clear at this point why a significant difference was observed only for *S. haematobium* and not for *S. mansoni*. Furthermore, a similar discrepancy was observed for CCR after multiple treatments (82.5% versus 68.7%, p = 0.04) but not for CERR (96.9% versus 94.6%, p = 0.05). This could suggest that pretreatment infection intensity alone cannot explain the influence of mixed *Schistosoma* infection on the efficacy of praziquantel treatment. Further research into the mechanisms by which mixed infections can shape treatment efficacy is needed.

No significant association was found between post-treatment reinfection with *Schistosoma* spp and baseline *Schistosoma* mixed infection status. This is in line with findings from other few studies which assessed the effect of mixed infection with *S. mansoni* and *S. haematobium* on reinfection patterns [13–15]. These findings are unexpected since mixed infection is commonly associated with higher infection intensity, which in turn, is associated with higher risk of reinfection. This suggests that infection intensity alone is not sufficient to explain the mechanism behind the association between *Schistosoma* mixed infection and reinfection patterns.

Younger age group (<16 years) was significantly associated with lower treatment efficacy against *S. haematobium* and higher reinfection rate with *S. haematobium* compared to >16 years age group. Our results are in accordance with previous studies showing that immunity against schistosomiasis, which is acquired with increasing age, enhances the efficacy of praziquantel [31]. As in other studies (see [12] for review) which explored reinfection patterns, the young age of reinfection found in our study can be explained by increased exposure and/or poor immune protection in children. Unfortunately, data on water contact and immunity were not collected in this study and their impact on observed reinfection could not be explored. Our results highlight the need for repeating praziquantel treatment in school aged children of our study area. Further studies are warranted to better understand why similar differences in age-related treatment efficacy or reinfection were not found for *S. mansoni*.

Although our study has the merit of addressing research questions on which there is little data in the DRC, it is important to point out certain limitations of the study. Firstly, nine weeks of post-treatment follow up does not allow to capture long-term outcomes in terms of both treatment efficacy and reinfection. A longer follow period would be more appropriate to study schistosomiasis reinfection patterns. Second, sensitivity of microscopy decreases when infection intensity is light, which is the case after treatment [32]. It is therefore possible that some light Schistosoma infections have been missed. Using a more sensitive diagnostic test would help refining the observed results.

## Conclusion

Our results indicate an overall acceptable efficacy of praziquantel treatment in our study area, with a shift of the relative composition of *Schistosoma* species after treatment. However, mixed *Schistosoma* infections alter the effect of both single and cumulative praziquantel treatment against *S. haematobium*. There was no evidence of an effect of pretreatment mixed infection status on reinfection patterns. Further studies are needed to better understand how multiple Schistosoma infections affect treatment efficacy and reinfection and the implications for disease control.
